# Identification of risk factors for hypertension in overweight and obese people and analysis of risk factor interactions: an R-based analysis

**DOI:** 10.3389/fcvm.2023.1180698

**Published:** 2023-10-31

**Authors:** LuWei Li, SiShuai Cheng, GuoQuan Xu

**Affiliations:** ^1^ Department of Rheumatology and Immunology, The First People’s Hospital of Nanning, Nanning, China; ^2^ School of Clinical Medicine, Guilin Medical University, Guilin, China; ^3^ Department of Urology, The First People’s Hospital of Qinzhou, Qinzhou, China

**Keywords:** overweight and obese, hypertension, risk factor, interaction, models

## Abstract

**Objective:**

This study identified the independent risk factors for hypertension in overweight and obese people and also analyzed the interaction between the risk factors.

**Methods:**

A total of 5,098 overweight and obese people were enrolled in this study. First, the clinical metabolic characteristics of hypertension and control groups were compared. The logistic regression (LR) and classification and regression trees (CRT)-based decision tree (DT) models were used to screen the independent risk factors for hypertension in overweight and obese people. The multiplicative and additive scale analyses were used to analyze the two risk factors with interaction from the perspective of statistics and biological interaction. Finally, the receiver operating characteristic (ROC) and calibration curves were used to analyze the accuracy and identification ability of the LR and DT models.

**Results:**

Age, UA, FPG, SBP, Cr, AST, TG, and FPG were higher in the hypertension group than in the control group (*P* < 0.05). The results of LR revealed that NAFLD, FPG, age, TG, LDL-c, UA, and Cr were positively correlated with hypertension in overweight and obese people, and GFR was negatively correlated with hypertension in overweight and obese people (*P* < 0.05). The DT model suggested that the risk factors of age, FPG, and UA interacted with each other. The multiplicative single and multiple factor analysis for FPG + UA, age + UA, age + FPG revealed a positive multiplicative interaction (*P* < 0.05, *B* ≠ 0, *OR* > 1). The additive single and multiple factor analysis for age + UA indicated a positive additive interaction. The ROC and calibration curve analysis indicated that the CRT decision tree, FPG + UA, age + UA, and age + FPG have certain accuracy and discrimination ability.

**Conclusion:**

The independent risk factors for hypertension in overweight and obese people included NAFLD, FPG, age, TG, LDL-c, UA, and Cr. Among these, age + UA exhibited synergistic interaction, thereby providing a reference for the prevention and control of hypertension in overweight and obese people.

## Introduction

Hypertension is a common chronic disease worldwide that often leads to cardiovascular and brain complications. According to the Chinese guidelines for the prevention and treatment of hypertension (1), the number of patients with hypertension is increasing every year in China. The 2020 International Society of Hypertension guidelines (2) also mentioned that in spite of the measures adopted, the adverse effects of hypertension and cardiovascular disease persist in the world. Overweight and obesity are risk factors for hypertension (3), and a gradual increase in body mass index (BMI) is observed in such individuals. The risk of hypertension in overweight and obese people is 1.16–1.28 times higher than that of normal-weight people (4). Relevant studies have shown (5) that the risk of hypertension in overweight people is 3–4 times higher than that in normal-weight people, indicating that overweight and obese people are prone to hypertension.

According to research by Zhang et al. (6), the prevalence of obesity-related hypertension among Chinese adults aged 45 and above is 22.7%, affecting approximately 120 million people. Among individuals aged 45–54, 55–64, 65–74, and ≥75 years, the prevalence rates of obesity-related hypertension are 16.7%, 24.3%, 27%, and 26.7%, respectively. The obesity prevalence rates in these age groups are also 16.7%, 24.3%, 27%, and 26.7%, respectively. In comparison, for other populations such as the United States, the prevalence of obesity and hypertension is even more pronounced. The proportion of overweight or obese adults in the U.S. population is as high as 70.7%, with a hypertension prevalence rate of 1.21% among adults residing in medium to small metropolitan statistical areas (MSAs), and a prevalence rate of 1.06% for adults residing in non-MSAs. Furthermore, at least 75% of hypertension incidence is directly linked to obesity (7–9). Previous studies have focused on all aspects of hypertension in the general population. However, the metabolic characteristics of overweight and obese people differ from those of the general population. Therefore, the independent risk factors for hypertension and their interactions with each other will differ among such populations. This study screened the independent risk factors for hypertension in overweight and obese people and explored the influence of risk factor interaction on hypertension to provide a reference for the prevention and treatment of hypertension from the perspective of etiology.

## Methods

### Study participants

In this cross-sectional study, patients who were evaluated at the physical examination department of the Affiliated Hospital of Guilin Medical University from August 2019 to November 2019 were selected. The patients signed an informed consent form, and the study was approved by the ethics committee of Guilin Medical University (approval number: GLMU1A2019064). Inclusion criteria for study participants: BMI ≥ 24 kg/m^2^; individuals willing to voluntarily participate in the study. Patients aged <18 years, pregnant women, and those with major diseases, such as malignant tumors, were excluded. A total of 5,098 patients with complete data were included in the study. The hospital had 84,000 discharges that year, and the sample size for this study is 5,098, accounting for 6.07% of the total. The age of the patients ranged from 18 to 85 years, with the average age being 44.92 ± 11.75 years. Of them, 3,609 were men aged 43.83 ± 11.72 years on average, accounting for 70.8% of the patients; moreover, 1,489 were women aged 47.54 ± 11.41 years on average, accounting for 29.2% of the patients. A total of 1,277 patients aged 50.55 ± 11.19 years on average were overweight and obese with hypertension; of these, 909 were men (71.1%), and 368 were women (28.9%). Moreover, 3,821 patients aged 43.03 ± 11.33 years on average were overweight and obese but without hypertension; of these, 2,700 were men (70.7%), and 1,121 were women (29.3%).

### Data collection

This study is conducted by professional in-service medical examination physicians who have undergone standardized training in the physical examination center for testing and data collection. During the physical examination of the patients, trained physicians evaluated their characteristics, including age, nationality and marital status, disease history, hypertension, nonalcoholic fatty liver disease (NAFLD), malignant tumor, severe liver and kidney function injury, relevant medication history, personal history, and family history.

### Physical measurement and measurement methods

The physical examination physicians used a SK-CK ultrasonic examination instrument (Shenzhen, China) to measure the height and weight of the patients. The height measurement was accurate to 0.1 cm, whereas the weight measurement was accurate to 0.1 kg. BMI was calculated using the following formula: BMI = weight (kg)/height (m^2^). The patients were instructed to rest quietly for 5–10 min and to adopt a sitting position. Subsequently, the blood pressure of the right upper arm was measured with an accuracy of 1 mmHg (1 mmHg = 0.133 kPa).

### Laboratory inspection and measurement methods

All the patients were instructed to have a light diet the day before the examination, and elbow venous blood (5 ml) was collected on an empty stomach on the morning of the examination day. The Roche's CobasC501 fully automatic biochemical analyzer (Roche Pharmaceutical Co. Ltd. matching reagent) was used to detect the biochemical indexes of fasting blood glucose (FPG), uric acid (UA), triglyceride (TG), total cholesterol (TC), low-density lipoprotein cholesterol (LDL-c), high-density lipoprotein cholesterol (HDL-c), creatinine (Cr), blood urea nitrogen (BUN), alanine aminotransferase (ALT), and aspartate aminotransferase (AST). Glomerular filtration rate (GFR) was calculated, and biochemical indicators are accurate to 2 decimal places. This study used the simplified Modification of Diet in Renal Disease (MDRD) formula that was improved by the Chinese: GFR (ml/[min × 1.73 m^2^]) = 186 × (Cr [mg/dl])^−1.154^ × (age)^−0.203^ × (0.742 women). The diagnosis of NAFLD was confirmed by an ultrasound specialist used the Hitachi ARIETTA 70 high-end ultrasound.

### Diagnostic criteria

The following diagnostic criteria of hypertension were defined in accordance with the global hypertension practice guide of the 2020 International Society of Hypertension (2): systolic blood pressure ≥140 mmHg and/or diastolic blood pressure ≥90 mmHg. The patients who had a previous diagnosis of hypertension and were taking medicines were also considered to have hypertension, although their blood pressure was normal. The following diagnostic criteria of dyslipidemia were defined in accordance with the 2018 criteria of the American Association of Clinical Endocrinologists and American College of Endocrinology (AACE/ACE) for managing dyslipidemia (10): TC ≥ 6.2 mmol/L; TG ≥ 2.3 mmol/L; LDL-c ≥ 4.1 mmol/L; or HDL-c < 1.0 mmol/L. According to the diabetes prevention guideline (version 2, 2020 edition) in China (11), the diagnostic standard of diabetes is FPG ≥7.0 mmol/L. The diagnostic standard of abnormal glucose tolerance was 6.1 mmol/L ≤ FPG < 7.0 mmol/L. Both diabetes and glucose intolerance disorders were diagnosed on the basis of an FPG value of >6.1 mmol/L. According to the guidelines for the diagnosis and treatment of NAFLD (2018 update) (12), the working definition of the disease is as follows: the results of imaging examinations, such as color Doppler ultrasound and CT, are similar to alcoholic liver disease; however, the patient does not have excessive drinking history. According to the 2019 guidelines for primary diagnosis and treatment of obesity (13), patients with BMI ≥ 24 kg/m^2^ are considered overweight and obese. According to the 2019 guidelines for the diagnosis and treatment of hyperuricemia and gout in China (14), hyperuricemia is defined as uric acid >420 µmol/L (both men and women). Fasting blood BUN ≥ 7.1 mmol/L was considered high BUN, while the fasting blood Cr level ≥110 µmol/L in men and ≥93 µmol/L in women was defined as hypercreatinine. Fasting blood ALT ≥ 40 U/L and fasting blood AST ≥ 40 U/L were considered high.

### Statistical analysis

MedCalc19.0.4, SPSS 26.0, and Rx64 4.0.3 were used for statistical analysis. First, clinical data on relevant risk factors were analyzed in hypertension and control (nonhypertension) groups, and the logistic regression (LR) and classification and regression trees (CRT) models of hypertension in overweight and obese people were established. The relevant independent influencing factors and those with interaction were screened, and the influencing factors with interaction were substituted into the product term of LR for the single factor and multiple factors multiplicative interaction test. For an assumption of 0.05 as the test level, the product term coefficient *B* of the LR model ≠ 0 [or the confidence interval of the odds ratio (*OR*) value does not include 1] and *P* < 0.05 indicated a multiplicative interaction between the two factors. *OR* > 1 indicated positive multiplicative interaction, whereas *OR* < 1 indicated negative multiplicative interaction. The receiver operating characteristic (ROC) and calibration curves of LR factors and CRT decision tree were used to analyze the accuracy and discrimination ability. The ggthemes program package was used for the visual analysis of additive interaction between relevant influencing factors. Three indicators, namely *RERI*, *AP*, and *SI*, were used to evaluate the meaningfulness of the additive interaction. No additive interaction between two factors was indicated by the confidence interval of 0 for *RERI* and *AP* and the confidence interval of 1 for *SI*. Contrasting results indicated an additive interaction.

## Results

### Clinical data analysis of hypertension and control groups

**Figure 1:**
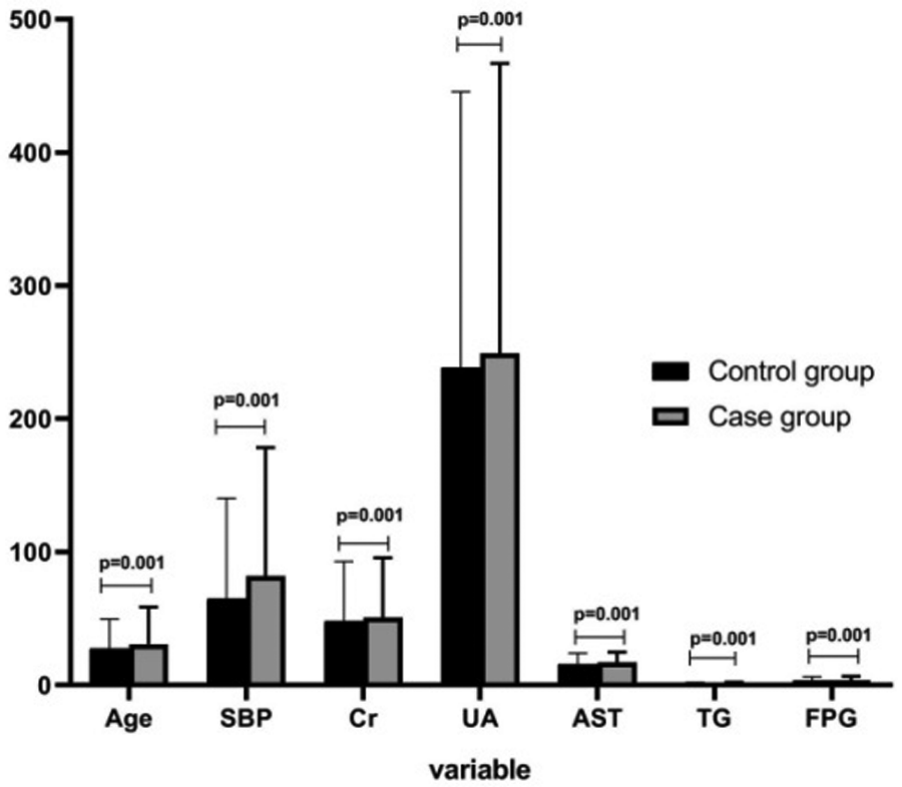
Comparison of influencing factors for hypertension in overweight and obese people between hypertension and control groups.

The overweight and obese patients with hypertension were defined as the hypertension group, and the overweight and obese patients without hypertension were defined as the control group. The analysis of clinical data revealed that age, UA, FPG, SBP, Cr, AST, TG, and FPG in the hypertension group were higher than those in the control group (*P* < 0.05; Figure 1).

### Logistic regression model construction

The risk factors were converted from continuous to classified variables. The risk factors were divided into two groups on the basis of their critical value. The value above the critical value was assigned as 1, and the value below the critical value was assigned as 0. The age boundary value was determined on the basis of the average age of overweight and obese patients with hypertension and without hypertension; 46 years was considered the average age, and the value ≥46 years was assigned as 1, whereas the value <46 years was assigned as 0 (Table 1).

**Table 1:** Main variables and assignment of influencing factors for hypertension in overweight and obese people.

Variable	Assignment
Hypertension	NO = 0, YES = 1
MAFLD	NO = 0, YES = 1
UA (μmol/L)	<420 = 0; ≥420 = 1
Age (years)	<46 = 0, ≥46 = 1
Gender	Female = 0, Male = 1
FPG (mmol/L)	<6.1 = 0; ≥6.1 = 1
ALT (U/L)	<40 = 0; ≥40 = 1
AST (U/L)	<40 = 0; ≥40 = 1
TG (mmol/L)	<2.26 = 0; ≥2.26 = 1
TC (mmol/L)	<6.22 = 0; ≥6.22 = 1
LDL-c (mmol/L)	<4.14 = 0; ≥4.14 = 1
HDL-c (mmol/L)	<1.04 = 0; ≥1.04 = 1
BUN (mmol/L)	<7.1 = 0; ≥7.1 = 1
GFR (ml/(min × 1.73 m^2^))	<90 = 0; ≥90 = 1
Cr (μmol/L)	Female: <93 = 0; ≥93 = 1
	Male: <110 = 0; ≥110 = 1

Overweight and obese patients with hypertension were assigned as 1, and overweight and obese patients without hypertension were assigned as 0. A univariate LR model was constructed by considering the presence or absence of hypertension as the dependent variable, and sex, age, NAFLD, UA, FPG, GFR, TG, TC, LDL-c, HDL-c, Cr, BUN, ALT, and AST as the independent variables. The results revealed that NAFLD, FPG, age, TG, TC, LDL-c, UA, and Cr were positively correlated with hypertension in overweight and obese patients, and GFR was negatively correlated with hypertension in overweight and obese patients (*P* < 0.05). The significant factors in the univariate logistic analysis were used to perform the multivariate LR analysis. The results revealed that NAFLD, FPG, age, TG, LDL-c, UA, and Cr were negatively correlated with hypertension in overweight and obese patients, and GFR was positively correlated with hypertension in overweight and obese patients (*P* < 0.05).

### Decision tree model construction

**Figure 2:**
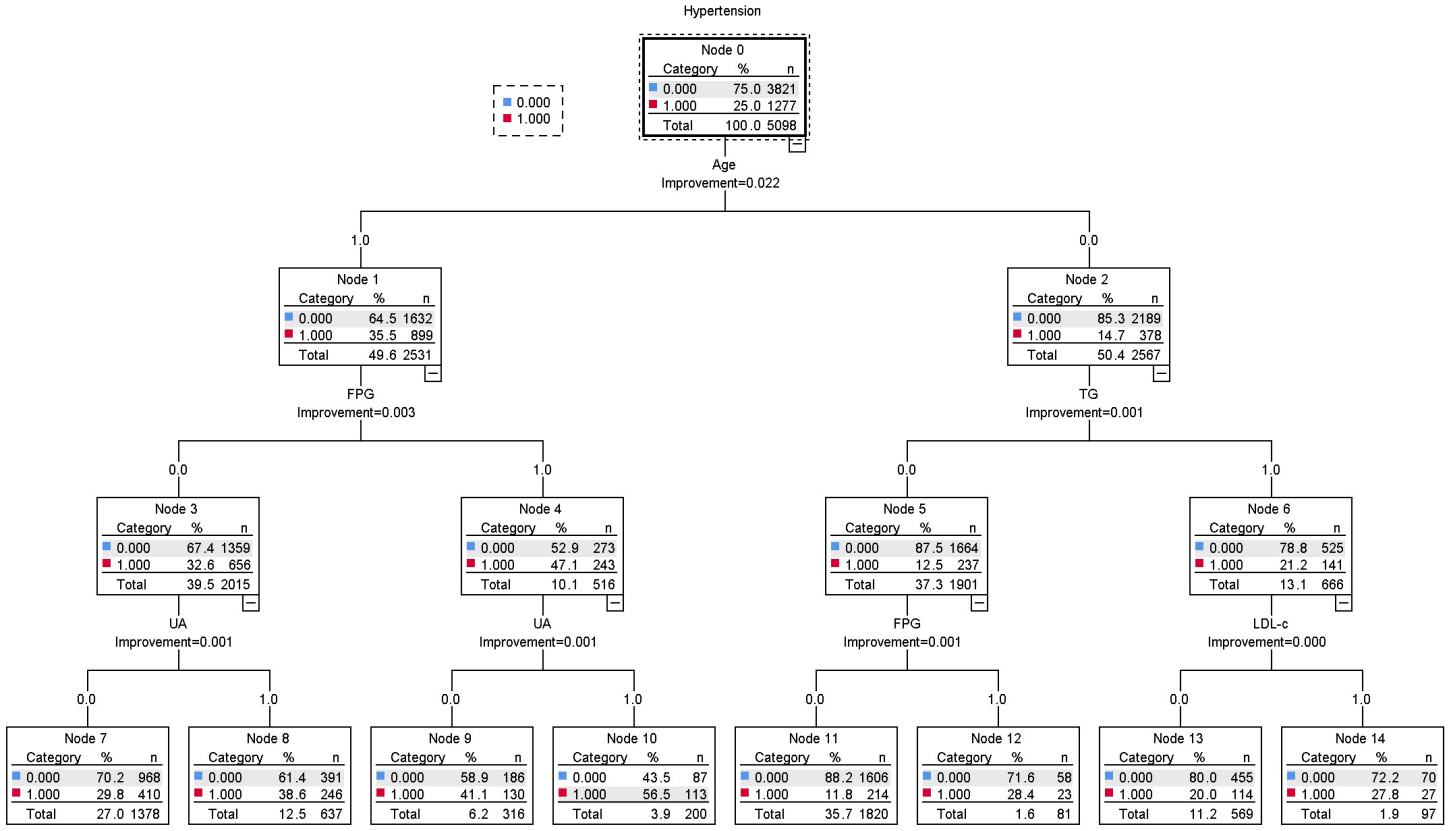
Decision tree model of influencing factors for hypertension in overweight and obese people.

Hypertension was considered the dependent variable, and the screening of NAFLD, FPG, Age, TG, LDL-c, UA, Cr, and GFR as independent variables was performed by the LR model. The classification and regression trees (CRT) method was used to establish the decision tree (DT) model. The DT was set to three layers, and the tree was pruned to avoid overfitting. The results revealed that age, FPG, UA, TG, and LDL-c were the risk factors for hypertension in overweight and obese patients. The results suggested a possible interaction between age, FPG, and UA (Figure 2).

### Multiplicative interaction analysis

The LR product term was used to analyze the multiplicative interaction of age, FPG, and UA screened by the DT model. The multivariate analysis included NAFLD, FPG, age, TG, LDL-c, UA, Cr, GFR, and other influencing factors screened by the LR model as confounding factors. The results indicated that FPG + UA, age + UA, and age + FPG had positive multiplicative interaction (*P* < 0.05, *B* ≠ 0, and *OR* > 1; Table 2).

**Table 2:** Single and multiple factor multiplicative interaction analysis of risk factors for hypertension in overweight and obese people.

Multiplicative interaction		Number of people	Single factor analysis		*P* value	Multivariate analysis		*P* value
			*B*	*OR* value (95% CI)		*B*	*OR* value (95% CI)	
FPG	UA							
+	−	406	0.941	2.564 (2.166–3.035)	0.001	0.533	1.704 (1.422–2.042)	0.001
−	+	1,570	0.340	1.406 (1.234–1.600)	0.001	0.266	1.305 (1.129–1.509)	0.001
+	+	264	1.169	3.221 (2.508–4.137)	0.001	0.729	2.073 (1.591–2.702)	0.001
Age	UA							
+	−	1,694	1.160	3.190 (2.783–3.655)	0.001	1.049	2.856 (2.468–3.304)	0.001
−	+	997	0.340	1.406 (1.234–1.600)	0.001	0.266	1.305 (1.129–1.509)	0.001
+	+	837	1.006	2.735 (2.341–3.194)	0.001	0.732	2.080 (1.759–2.459)	0.001
Age	FPG							
+	−	2,015	1.160	3.190 (2.783–3.655)	0.001	1.049	2.856 (2.468–3.304)	0.001
−	+	154	0.941	2.564 (2.166–3.035)	0.001	0.533	1.704 (1.422–2.042)	0.001
+	+	516	1.116	3.054 (2.535–3.679)	0.001	0.958	2.606 (2.150–3.158)	0.001

### Model validation analysis

**Figure 3:**
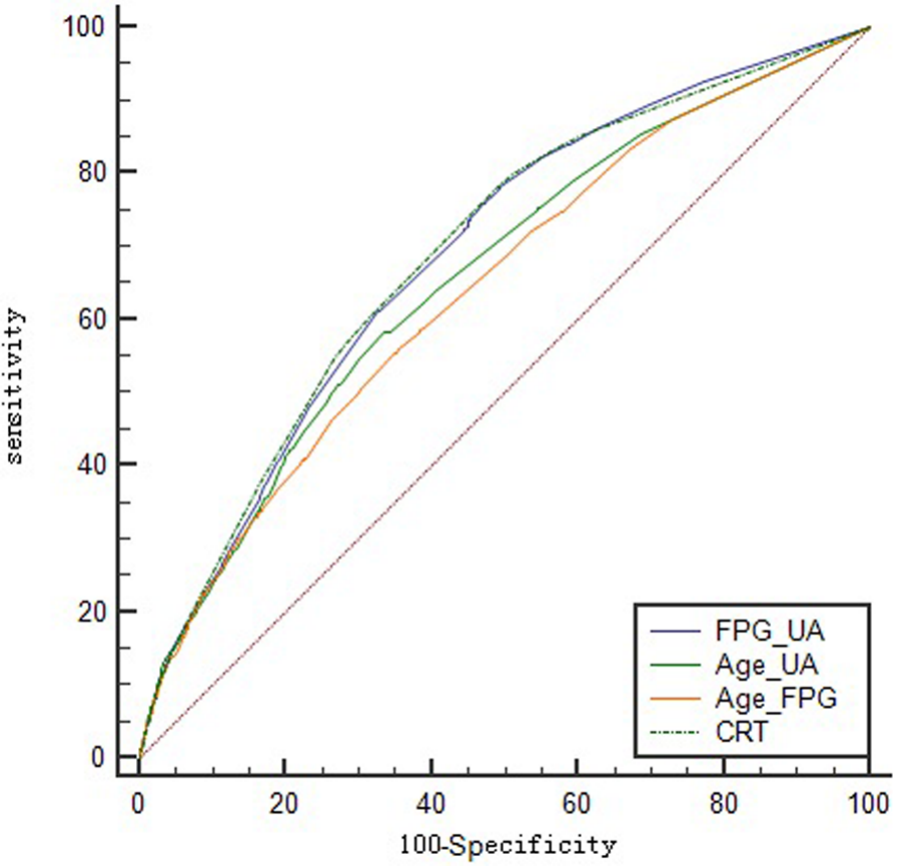
Logistic regression and classification tree ROC curve of hypertension in overweight and obese people.

**Figure 4:**
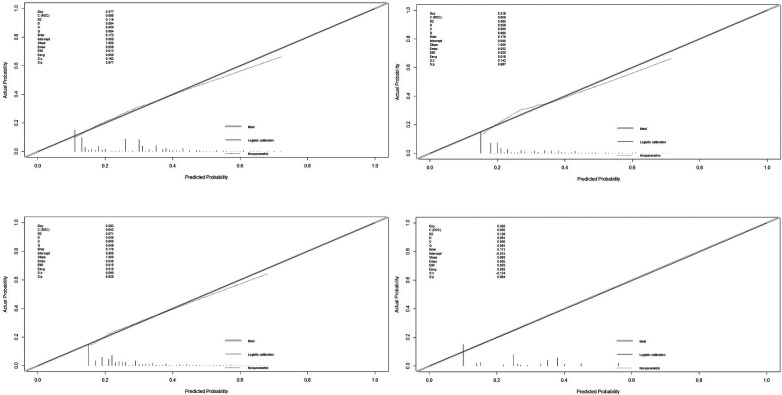
Logistic regression and classification tree calibration curve of hypertension in overweight and obese people (FPG + UA, Age + UA, Age + FPG, and CRT).

The ROC and calibration curves of the patients were constructed using the predictive variables obtained by the CRT DT and LR multivariate analysis of FPG + UA, age + UA, and age + FPG as variables, and the presence or absence of hypertension in overweight and obese patients as categorical variables. The results indicated the accuracy and discrimination ability of the CRT DT, FPG + UA, age + UA, and age + FPG models (Figures 3, 4).

### Additive interaction analysis

**Figure 5:**
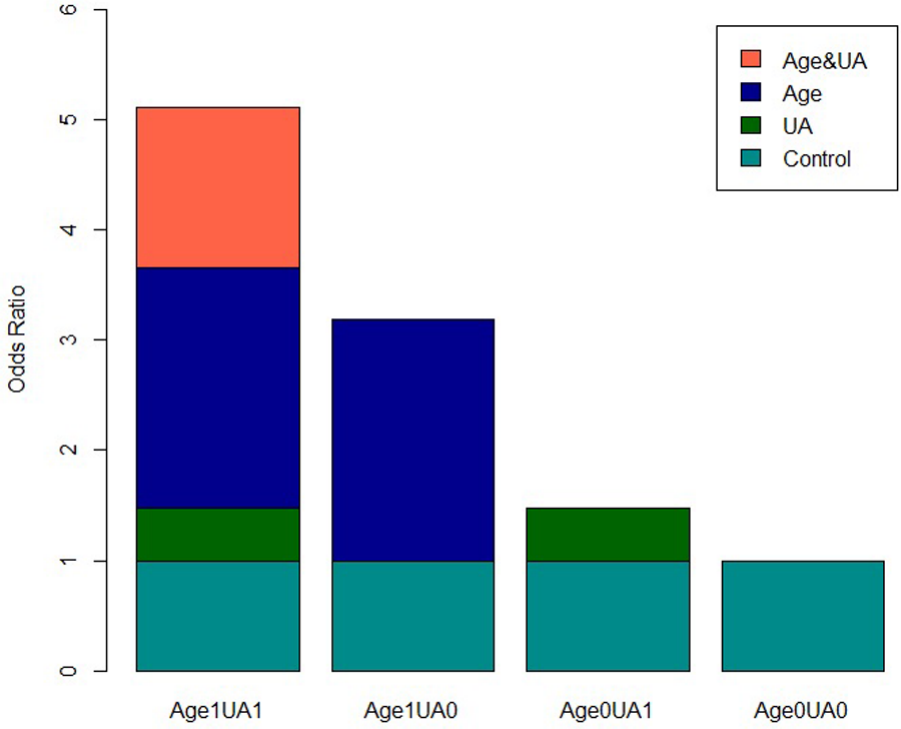
Visual analysis of single factor additive interaction of risk factors for hypertension in overweight and obese people.

**Figure 6:**
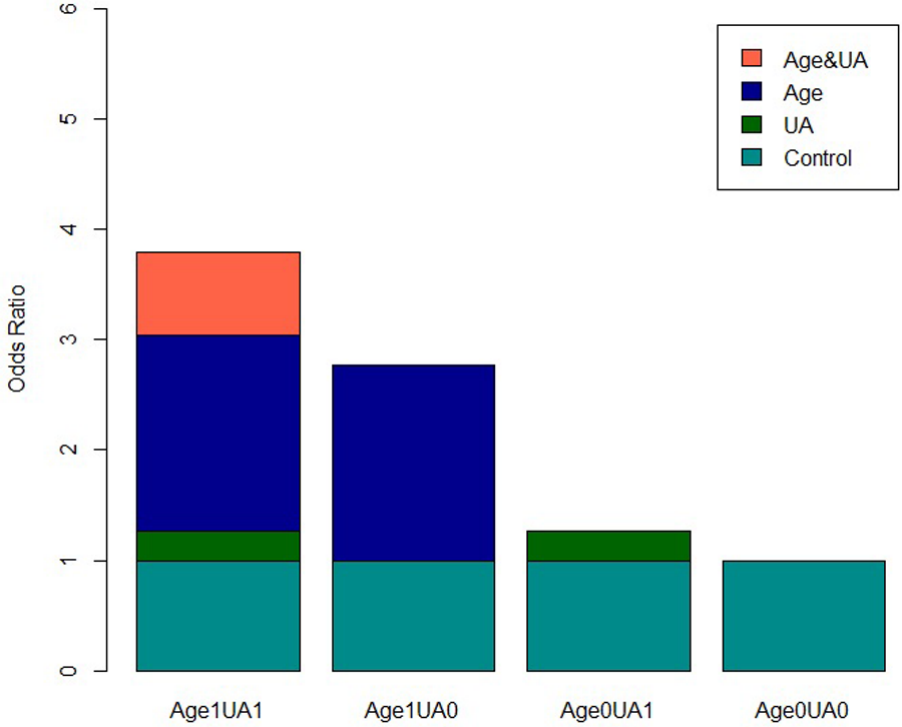
Visual analysis of multivariate additive interaction of risk factors for hypertension in overweight and obese people.

The R was used to visually analyze the additive effect of single and multiple factors for FPG + UA, age + UA, and age + FPG. The multivariate analysis included NAFLD, FPG, age, TG, LDL-c, UA, Cr, GFR, and other influencing factors screened by the LR model as confounding factors. The results indicated that age + UA univariate and multivariate analyses (the confidence interval of *RERI* and *AP* does not include 0, and the confidence interval of *SI* does not include 1) and FPG + UA univariate analysis exhibited additive interaction; however, FPG + UA multivariate analysis and age + FPG univariate and multivariate analyses did not exhibit additive interaction (Tables 3, 4; Figures 5, 6).

**Table 3:** Evaluation indexes of additive interaction of risk factors for hypertension in overweight and obese people.

Additive interaction evaluation index	Single factor analysis	Multivariate analysis
FPG + UA		
RERI (95% CI)	1.188 (0.134–2.242)	0.794 (−0.036–1.625)
AP (95% CI)	0.307 (0.096–0.519)	0.273 (0.040–0.505)
SI (95% CI)	1.710 (1.095–2.671)	1.714 (0.991–2.961)
Age + UA		
RERI (95% CI)	1.458 (0.646–2.269)	0.758 (0.101–1.415)
AP (95% CI)	0.285 (0.156–0.414)	0.199 (0.046–0.352)
SI (95% CI)	1.548 (1.217–1.969)	1.371 (1.041–1.807)
Age + FPG		
RERI (95% CI)	1.200 (−0.087–2.488)	0.917 (−0.223–2.057)
AP (95% CI)	0.218 (0.009–0.426)	0.189 (−0.023–0.403)
SI (95% CI)	1.363 (0.970–1.915)	1.315 (0.930–1.859)

**Table 4:** Single and multiple factor additive interaction analysis of risk factors for hypertension in overweight and obese people.

Additive interaction		Number of people	Single factor analysis	Multivariate analysis
			*OR* value (95% CI)	*OR* value (95% CI)
FPG	UA			
+	−	406	2.334 (1.872–2.910)	1.996 (1.590–2.507)
−	+	1,570	1.337 (1.157–1.546)	1.115 (0.957–1.300)
+	+	264	3.860 (2.983–4.995)	2.907 (2.218–3.808)
Age	UA			
+	−	1,694	3.187 (2.662–3.815)	2.769 (2.300–3.334)
−	+	997	1.470 (1.180–1.831)	1.269 (1.012–1.592)
+	+	837	5.115 (4.181–6.258)	3.798 (3.055–4.722)
Age	FPG			
+	−	2,015	2.983 (2.572–3.460)	2.923 (2.503–3.414)
−	+	154	2.318 (1.596–3.365)	1.987 (1.359–2.907)
+	+	516	5.502 (4.469–6.773)	4.828(3.892–5.990)

## Discussion

As the population of individuals with hypertension related to overweight and obesity continues to grow (6), corresponding research has also been increasing. This study aims to investigate the factors influencing hypertension in overweight and obese people and further explore the depth of their interactions using multiplicative and additive models. The goal is to provide valuable insights for clinical research. First, the clinical metabolic characteristics of hypertension and control groups were compared. The results indicated that age, UA, FPG, SBP, Cr, AST, TG, and FPG of the hypertension group were higher than those of the control group. Second, the relevant risk factors for hypertension in overweight and obese people were screened. This study referred to the relevant program statements prepared by Xu et al. (15) and used dichotomous variables to explore the independent effects of risk factors and analyze the interaction. An LR model of hypertension in overweight and obese people was constructed, and a single factor and multiple factor analysis were performed to screen the independent influencing factors. The results indicated that NAFLD, FPG, age, TG, LDL-c, UA, and Cr were positively correlated with hypertension in overweight and obese patients, and GFR was negatively correlated with hypertension in overweight and obese patients. The DT analysis was performed using the CRT method, and the results revealed that the risk factors for hypertension in overweight and obese patients included age, FPG, UA, TG, and LDL-c; these results are similar to those of previous studies (16, 17). The CRT DT model also suggested that the prevalence rate of hypertension in overweight and obese patients aged ≥46 years and with FPG ≥ 6.1 mmol/L was 47.1%. However, the prevalence rate of hypertension in overweight and obese patients aged ≥46 years, with FPG ≥ 6.1 mmol/L, and with UA ≥ 420 µmol/L increased to 56.5%, suggesting that the interaction effect of age, FPG, and UA plays a crucial role in the prevalence of hypertension in overweight and obese people.

Interaction in the multivariate statistical analysis means that the effect of a certain factor varies with the level of other factors. When two factors coexist, the effect is not equal to the sum (additive interaction) or the product (multiplicative interaction) of the two factors acting alone. In this study, the multiplicative interaction was analyzed by constructing the LR product term by performing the single and multiple factor analysis of the relevant influencing factors. The multiple factor analysis included all the influencing factors for hypertension in overweight and obese people that were screened by the LR model as confounding factors. The results suggested that FPG + UA, age + UA, age + FPG, and other combinations have positive multiplicative interaction in the univariate and multivariate analyses (*P* < 0.05, *B* ≠ 0, *OR* > 1). Compared with the independent existence of related factors, the combined existence will increase the prevalence rate of hypertension by 2–3 times.

Biological interaction is the combined effects, including synergism and antagonism, of two factors on biological mechanisms. Rothman (18) proposed that the evaluation of biological interaction should be based on the additive scale. In this study, we conducted a quantitative and visual analysis of the additive interaction between hypertension risk factors in overweight and obese people. The results indicated that the univariate and multivariate analyses of age + UA had additive interaction (the confidence interval of *RERI* and *AP* did not include 0, and the confidence interval of *SI* did not include 1). The quantitative analysis suggested that the prevalence of hypertension in overweight and obese patients increased 4 or 5 times when the two factors were combined.

The verification of the accuracy and discrimination ability of the LR and CRT DT models indicated that the four models, FPG + UA, age + UA, age + FPG, and CRT, have obvious discrimination ability (*P* > 0.05), and the ROC curve indicated that the four models have certain accuracy.

This study suggested that age is a crucial independent risk factor for hypertension in overweight and obese people. This finding is similar to those of previous studies. For example, Batte (19) and others suggested that overweight, obesity, and age >40 years are statistically significant predictors of hypertension. Ahammed (20) and others also reported that age has a significant impact on hypertension. The International Society of Hypertension 2020 guidelines (2) proposed that age >40 years can predict hypertension. Therefore, age is an independent risk factor for hypertension in overweight and obese people and may be related to impaired vascular endothelial function, oxidative stress, nitric oxide deficiency, and arterial remodeling (21, 22).

This study also suggested that hyperglycemia is a crucial risk factor for hypertension which is in agreement with the results of several studies. Insulin resistance (23) and chronic low-grade inflammation that may be partially mediated by insulin resistance (24) are the risk factors for hypertension in overweight and obese people. Hyperuricemia, as suggested by this study, is another important independent risk factor for hypertension in overweight and obese people (25), although the association mechanism between them remains unclear. Studies have demonstrated (26) that every 1% increase in serum UA level increases the risk of hypertension by 13%. Hyperuricemia, as an independent risk factor for hypertension in overweight and obese people, may be closely related to chronic kidney injury (27).

Big data statistical studies on hypertension in the Chinese population, such as the discussion on the prediction model and independent risk factors for hypertension in Central China by Ren et al. (28), Chen (29) and others, analyzed the population in northern China and constructed the corresponding prediction model of hypertension. These studies explored the independent risk factors but did not focus on the prevalence of hypertension in overweight and obese people and the interaction between relevant risk factors. Therefore, this study conducted multiplicative and additive interaction analyses of relevant risk factors. Both multiplicative and additive interactions were significant for patients aged ≥46 years with UA ≥ 420 µmol/L; the synergistic effect of the combination of the two risk factors has an important impact on the prevalence of hypertension in overweight and obese people. However, Kim (30) reported that the relationship between UA and hypertension often depends on age and sex. Therefore, for people aged above 46 years, it is crucial to control the level of UA for the prevention of hypertension.

### Limitations

This study has limitations. Firstly, the study is a single-center research conducted on a specific group of individuals undergoing health check-ups at our institution. The sample size is not extensive, and due to the nature of health check-up participants, detailed inquiries about general aspects like sleep, diet, and mental well-being, akin to what would be done with hospitalized patients, were not included. As a result, lifestyle factors such as sleep patterns, dietary habits, and calorie intake were not considered in the study. Moreover, the study's focus on health check-up participants precluded the ability to precisely determine the duration of hypertension, overweight, and obesity. The timeframe during which these health issues were present and their duration could not be established, which is also a limitation of this research. Finally, the study is confined to a single center, lacking validation of the research findings and based solely on health check-up data from a specific region, potentially limiting its applicability to a broader population.

Future research should encompass multi-center collaborations to gather more diverse samples. Additionally, incorporating detailed inquiries about medical history and lifestyle factors, including sleep, diet, and calorie intake, alongside the examination variables like systolic and diastolic blood pressure, cholesterol, and triglycerides, would enhance the research's depth. Furthermore, validation measures are necessary to augment the study's credibility.

## Conclusions

Independent risk factors for hypertension in overweight and obese people included NAFLD, FPG, age, TG, LDL-c, UA, and Cr. Among these, age + UA has a synergistic interaction, with the combined effect being four times higher than the single effect. The four models have obvious discrimination ability and certain accuracy. Therefore, older adults with hyperuricemia should monitor and control hypertension.

## Data Availability

The raw data supporting the conclusions of this article will be made available by the authors, without undue reservation.
